# Anisotropic dynamics of two-photon ionization: An attosecond movie of photoemission

**DOI:** 10.1126/sciadv.abl7594

**Published:** 2022-03-23

**Authors:** Alice Autuori, Dominique Platzer, Mariusz Lejman, Guillaume Gallician, Lucie Maëder, Antoine Covolo, Lea Bosse, Malay Dalui, David Bresteau, Jean-François Hergott, Olivier Tcherbakoff, Hugo J. B. Marroux, Vincent Loriot, Franck Lépine, Lionel Poisson, Richard Taïeb, Jérémie Caillat, Pascal Salières

**Affiliations:** 1 Université Paris-Saclay, CEA, CNRS, LIDYL,91191 Gif-sur-Yvette, France.; 2 Université de Lyon, Université Claude Bernard Lyon 1, CNRS, Institut Lumière Matière, 69622 Villeurbanne, France.; 3 Université Paris-Saclay, CNRS, Institut des Sciences Moléculaires d’Orsay,91405 Orsay, France.; 4 Sorbonne Université, CNRS, Laboratoire de Chimie Physique-Matière et Rayonnement, 75005 Paris, France.

## Abstract

Imaging in real time the complete dynamics of a process as fundamental as photoemission has long been out of reach because of the difficulty of combining attosecond temporal resolution with fine spectral and angular resolutions. Here, we achieve full decoding of the intricate angle-dependent dynamics of a photoemission process in helium, spectrally and anisotropically structured by two-photon transitions through intermediate bound states. Using spectrally and angularly resolved attosecond electron interferometry, we characterize the complex-valued transition probability amplitude toward the photoelectron quantum state. This allows reconstructing in space, time, and energy the complete formation of the photoionized wave packet.

## Teaser

Using attosecond electron interferometry, the photoemission process is imaged in space and time at the atomic level.

## INTRODUCTION

Photoemission has played a key role in the development of quantum physics theory. Besides its fundamental aspects, it has become a widespread technique for the investigation of matter, from chemical analysis in condensed matter to electronic structure determination in the gas phase. The development of bright sources of extreme ultraviolet (XUV) radiation such as synchrotrons has allowed the measurement of angular distributions of photoelectrons with high precision both in the laboratory and molecular frames. Pioneering complete experiments (*1*, *2*) gave access to the relative weights and phases of the partial waves contributing to the wave function of the liberated electron. These measurements, however, do not give access to photoemission in the time domain because they are performed independently for each final electron energy and thus lack the relative phase between the spectral components.

The advent of broadband coherent XUV sources based on high-order harmonic generation (HHG) from intense infrared (IR) pulses (*3*, *4*) has triggered the development of unique interferometric schemes to measure spectral phases, leading to unprecedented insight into the temporal dynamics of elementary processes in a broad range of chemical species with attosecond resolution (1 as =10^−18^ s). Both the streaking (*5*) and the Reconstruction of Attosecond Beating By Interference of Two-Photon Transitions (RABBIT) technique (*6*–*9*) were applied to revisit photoemission in the time domain, through the first measurements of attosecond photoemission delays in atoms (*10*, *11*), molecules (*12*, *13*), nanoparticles (*14*), liquids (*15*), and solids (*16*). Access to higher levels of details was gradually achieved using tunable sources (*17*–*21*) or by increasing the spectral resolution at detection with the Rainbow RABBIT technique (*22*–*25*). However, all the aforementioned studies relied on measurements averaged over the direction of photoemission. While attosecond photoelectron interferometry has been investigated in momentum space in earlier experiments, e.g., in (*26*, *27*), including the pioneering study (*28*), it is only recently that orientation-resolved spectral phase measurements could be performed, using cold target recoil ion momentum spectroscopy (COLTRIMS) (*29*–*34*) or velocity-map imaging spectroscopy (VMIS) (*35*–*38*). Resonant photoemission dynamics was studied angularly with RABBIT in the vicinity of, e.g., autoionizing states (*30*) or shape resonances (*34*). Using a two-color above-threshold interferometric scheme, autoionizing dynamics in chiral molecules could be angularly resolved in the laboratory frame (*35*).

In the present study, we combine attosecond spectral interferometry with momentum spectroscopy to record the modulus and phase variations of the photoelectron quantum state with high spectral resolution and angular sensitivity. The potential of this complete quantum phase spectroscopy is demonstrated in the test case of two-photon XUV + IR photoionization of helium through the intermediate resonant states 1s3p and 1s4p. The resulting structured photoelectron wave packet is fully characterized by measuring quasi-continuously the spectral and spatial variations of the final quantum state over a 0.8-eV spectral range. This allows reconstructing the attosecond photoemission dynamics strongly affected by the sudden phase jumps of up to π rad measured in both dimensions.

## RESULTS

**Fig. 1.**
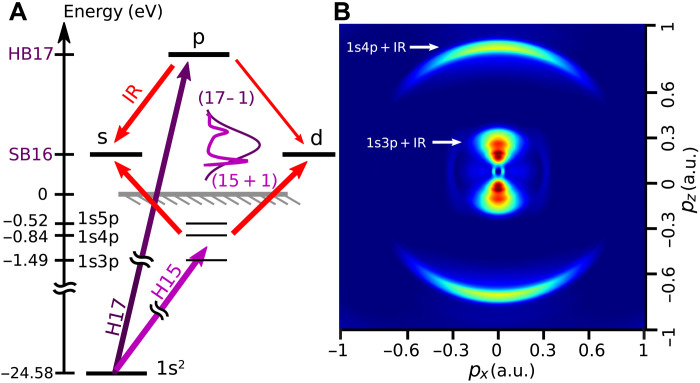
Principle of spectrally and angularly resolved photoelectron interferometry. (**A**) Helium is ionized with a comb of coherent odd harmonics of a titanium:sapphire laser combined with a weak fraction of the fundamental laser. The two-photon electron wave packet created in the continuum by absorption of H15 and an IR photon is highly structured because of the intermediate 1s3p and 1s4p resonant states. This wave packet, (15 + 1), is a coherent superposition of the s and d partial waves that are populated by the two-photon transition. A reference wave packet, (17 − 1), is created at the same energy by absorption of H17 and stimulated emission of an IR photon. (**B**) Momentum map of the delay-integrated SB16 from an Abel-inverted VMIS measurement. It exhibits two main spectral components centered around 1s3p + IR and 1s4p + IR.

The concept of the technique is illustrated in Fig. 1A, the corresponding experimental setup being detailed in the Supplementary Materials. We aim at fully characterizing the resonant (15 + 1) two-photon transition in He using a reference (17 − 1) transition. Their interference determines the photoelectron angular and spectral distribution in the sideband peak (SB16) that is measured by VMIS (see Fig. 1B). The SB16 intensity *I*_16_ thus depends on three parameters: (i) the XUV-IR delay τ, (ii) the photoelectron energy *E*, and (iii) the angle θ between the electron emission and the shared XUV-IR polarization axis *z*, in the generic form

$$I_{16}(\tau ;E,\theta )=A_{16}(E,\theta )+B_{16}(E,\theta )\text{cos}\;[2\omega_{0}\tau -C_{16}(E,\theta )]$$ (1)

where ω_0_ is the laser frequency. Our study focuses on the amplitude *B*_16_ and phase *C*_16_, of which the complex anisotropic photoemission dynamics is decoded. Both quantities are accessed through a Fourier transform of the three-dimensional (3D) spectrogram *I*_16_(τ; *E*, θ) with respect to τ at each sampled energy *E*. They are then calibrated to extract the intrinsic two-photon transition amplitude *M*(*E*, θ) associated with the probed (15 + 1) path independently of the characteristics of the XUV exciting pulses (see the Supplementary Materials).

**Fig. 2.**
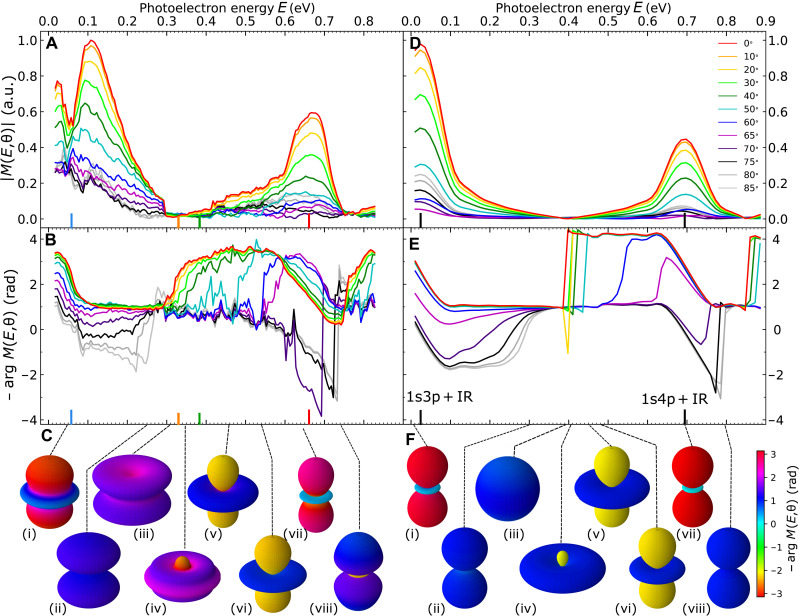
Probability amplitude associated with the investigated (15 + 1) two-photon transition. Spectral and angular variations of the modulus and phase of *M*(*E*, θ) resulting from a Rainbow RABBIT analysis of the measured (**A** and **B**, respectively) and simulated (**D** and **E**, respectively) spectrogram *I*_16_(τ; *E*, θ). The phases are shown with a negative sign for readability. Polar representation of *M*(*E*, θ) at specific energies labeled from (i) to (viii), where the θ-dependent radius and color correspond respectively to the modulus and phase from experimental measurements (**C**) and from TDSE simulations (**F**).

The modulus and phase of *M*(*E*, θ) obtained from the experimental data are shown in Fig. 2 (A and B). They display many structures related to the buildup of the wave packet through intermediate resonances. At *E* ≈ *E*_1s*n*p_ + ℏω_0_, the modulus is enhanced and a ≈π rad smooth spectral phase drop occurs, reminiscent of the observations of (*17*). In contrast, between resonances, the signal substantially drops and a sharp ≈π rad spectral phase jump occurs. A most remarkable feature is the strong angular dependence of both modulus and phase (jumps up to π rad) over the covered spectral range. This is highlighted by fast changes in shape and phase of the polar representation of *M*(*E*, θ), as shown in Fig. 2C for few selected representative energies.

To support the analysis of our measurements, we performed numerical simulations based on the time-dependent Schrödinger equation (TDSE) with a model potential for helium and pulse characteristics corresponding to the experimental ones. The results, shown in Fig. 2 (D and E), are in good agreement with the experimental ones, the latter showing slightly attenuated phase jumps that are down-shifted by ∼80 meV around *E* = 0.32 eV and a stronger angular dependence around the 1s4p resonance.

## DISCUSSION

The observed rich spectral and angular features are the signature of a structured anisotropic photoemission dynamics, fully encoded in the differential transition probability amplitudes *M*(*E*, θ). They can be understood through a partial-wave expansion of the latter

$$M(E,\theta )=M_{0}(E)Y_{00}(\theta )+M_{2}(E)Y_{20}(\theta )$$ (2)

where *Y*_𝓁0_ are the spherical harmonics and ℳ_𝓁_(*E*) are the matrix elements associated with the final angular momenta 𝓁.

**Fig. 3.**
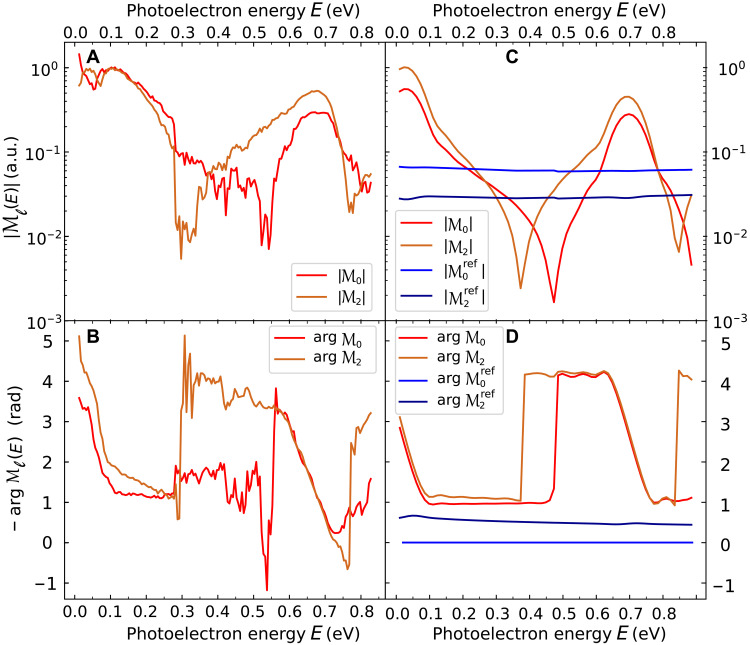
Partial-wave decomposition of the investigated two-photon transition. Modulus and phase of the partial-wave transition amplitudes ℳ_𝓁_(*E*) (𝓁 = 0 and 2) obtained from experiments (**A** and **B**, respectively) and from simulations (**C** and **D**, respectively). The simulated partial-wave amplitudes for the reference transition are also shown in (C) and (D), with $\text{arg}\;M_{0}^{\text{ref}}(E)$ taken as phase origin.

For monochromatic fields, in our spectral range where the XUV photon frequency Ω is nearly resonant with the 1s*n*p states, the partial amplitudes can be approximated by (*39*)

$$M_{\ell }(E)\approx \underset{\epsilon \to 0^{+}}{\text{lim}}\sum_{n=3}^{5}\frac{\langle 1sE\ell |z|1snp\rangle \langle 1snp|z|1s^{2}\rangle }{E_{1s^{2}}-E_{1snp}+\hbar \Omega +i\epsilon }$$ (3)

where ∣1s^2^〉 is the initial ground state of energy *E*_1s^2^_, ∣1s*E*ℓ〉 is the considered final partial wave with photoelectron energy *E* = *E*_1s^2^_ + ℏΩ + ℏω_0_. In our experiments and simulations, the finite duration/bandwidth of the XUV and IR fields results in a smoothing of the outcomes from Eq. 3 (*40*, *41*), which accounts for the spectral variations of ℳ_𝓁_(*E*) shown in Fig. 3 (C and D).

In contrast to the structureless (17 − 1) reference path, dominated by the isotropic s wave, the studied transition matrix element for each of the 𝓁 channels undergoes a series of π rad phase jumps accompanied by a strong modulus increase, around 0.06- and 0.71-eV photoelectron energies. They are reminiscent of a zero crossing of the denominator in Eq. 3, wherever Ω is resonant with one of the 1s*n*p (*n* = 3 and 4) levels. Furthermore, between consecutive resonances, two contributions with opposite signs dominate the sum and cancel each other at a given energy, depending on the relative values of the 𝓁-dependent numerators. This results in a local minimum in the transition amplitude and a very sharp π phase jump at 0.47 (0.37) eV for the s (d) channel. These 𝓁-specific cancelations are highly sensitive to the resonance energies and relative strengths. Their precise positions are also affected by the real-valued amplitudes associated with the “IR-first” two-photon paths (see fig. S3). Despite being relatively small, these background contributions become nonnegligible where the effective amplitude associated with all the other paths vanishes. They have no other consequence on our data analysis and interpretation. The cancelations are measured at 0.54 (0.30) eV for the s (d) channel in the experimental decomposition in Fig. 3 (A and B) that otherwise displays a good agreement with the theoretical prediction.

The relative imprint of these 𝓁 contributions over the whole energy range is clearly visible in the polar representation of the final quantum state in Fig. 2 (C and F), which again shows a good correspondence between experiments and simulations and slight shifts in energy. In the 1s3p resonance region, the dominant d wave results in a minimum and change of sign at an angle θ_0_ ≈ 60° (*37*, *42*) (red to cyan in plot i), close to the node of *Y*_20_(θ). As the energy increases, the d amplitude becomes less dominant, θ_0_ ⟶ 90° and the sign change disappears (ii). The sharp d-wave cancelation results in a (almost) spherical s state (iii), experimentally distorted by a residual ℳ_4_ contribution. Conversely, the s-wave cancelation leads to a typical d state (vi). Between these energies, the π relative phase of the s and d components produces a strong destructive interference at small angles, resulting in a doughnut shape (iv) evolving toward a d shape (v). In the 1s4p resonance region (vii and viii), one recovers the initial shapes (i and ii). A possible source of discrepancies between the measurements and the theoretical results is the absence of electron correlation (*43*, *44*) in our single-active electron model. This may notably affect the highly sensitive positions of the destructive interferences, but it has no practical consequence on our analysis.

Our measurements give direct access to the complete, angularly resolved dynamics of the two-photon transition leading to photoemission. A way of characterizing this dynamics is provided by the transition delay, a quantity specific to multiphoton processes defined as the local spectral derivative of the transition phase [see (*41*) and references therein], for each emission angle θ

$$\tau_{\text{tran}}(E,\theta )=\frac{\partial \text{arg}\;M(E,\theta )}{\partial E}$$ (4)

**Fig. 4.**
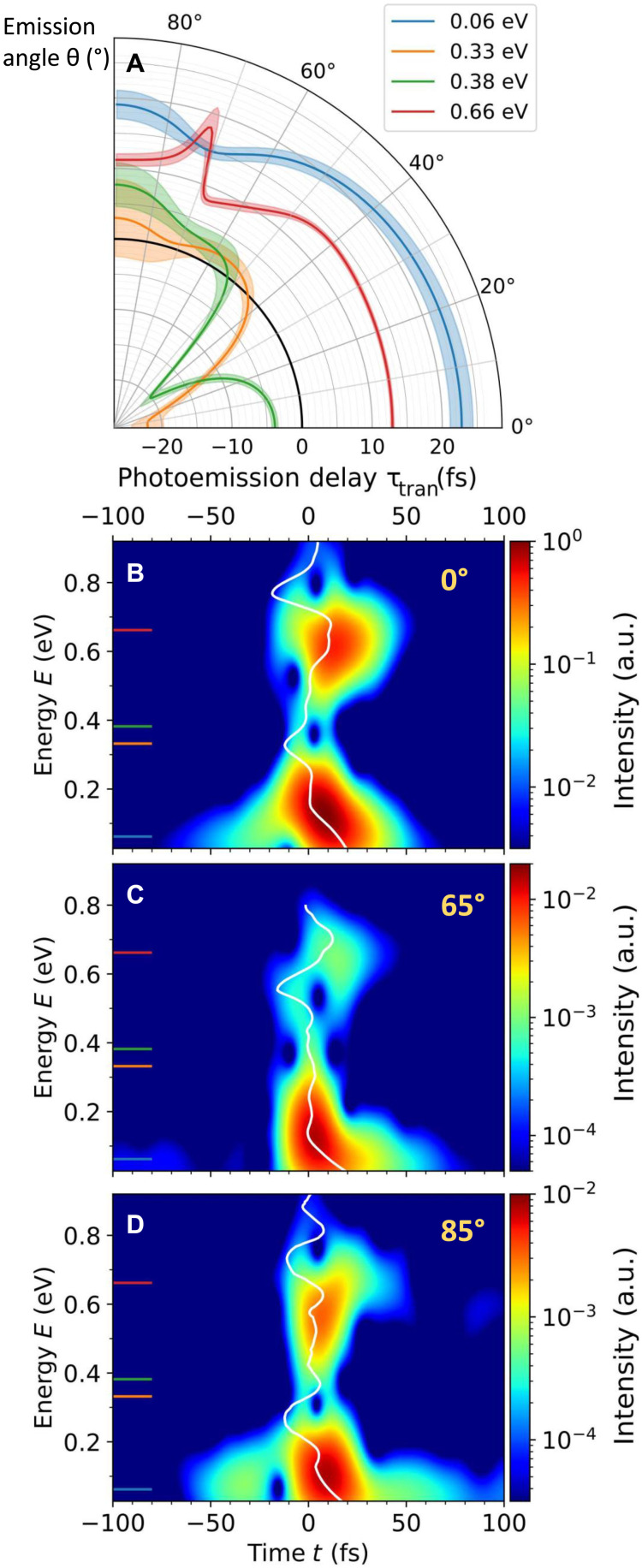
Photoemission dynamics retrieved from the experimental amplitudes *M*(*E*, θ). (**A**) Angular dependence of the photoemission delay τ_tran_ determined by linear fit over 40 meV around four representative energies indicated by color ticks in Fig. 2 and corresponding uncertainty given by the fit SD. (**B**, **C** and **D**) Spectrotemporal buildup of the process revealed by the spectrally gated ionization rates, obtained by Gabor analysis of *M*(*E*, θ) at three representative angles: 0, 65 and 85°, respectively. The transition delay τ_tran_ as a function of *E* is overlaid on each Gabor representation (white lines).

The results, shown in Fig. 4A and in the Supplementary Materials, reveal that, in the resonance regions, the delay is strongly positive and weakly anisotropic, while in the intermediate region, it is strongly negative and varies a lot with angle. The physical interpretation of this transition delay is straightforward at each resonance: It represents an effective time during which the electron is transiently trapped in the intermediate bound state, before completing the transition (*41*). Its effective value is bounded by the experimental duration of the IR, which acts as a temporal gate on the process. In the intermediate region, between resonances, where the delay takes both positive and negative values, the interpretation is not so intuitive. The dynamics there are exclusively shaped by destructive quantum interferences between the two-photon paths through the 1s3p and 1s4p intermediate states, respectively.

To get a deeper insight on how the different spectral components interfere in the temporal buildup of the photoelectron wave packet, we perform a Gabor analysis of the experimental *M*(*E*, θ) using a sliding 210-meV Gaussian gate as a balanced spectrotemporal resolution compromise. Moreover, the spectral gate is narrow enough to allow a direct connection between the obtained time-domain data and the atomic ionization rate (*45*). The results for three illustrative angles are shown in Fig. 4 (B to D), overlaid with τ_tran_ now as a function of *E*. As anticipated, the revealed dynamics appear to be globally “delayed” at resonances (i.e., around 0.06 and 0.66 eV). Apart from this, they are strongly shaped by the presence of the spectral phase jumps distributed over the covered range. They result in destructive interferences in the temporal profiles, appearing as “holes” in the Gabor transform. In particular, the holes in the intermediate region between resonances shift toward higher energies when θ increases, as they follow the spectral phase jump (e.g., from 0.33 eV at 0° to 0.59 eV at 65°). The spectrotemporal structuring of the dynamics therefore highly depends on the photoemission angle (see movies in the Supplementary Materials).

At each angle, the group delay τ_tran_ follows the apparent ridge line skirting around the holes in the ionization rate maps. This emphasizes the irrelevance of any attempt to intuitively interpret negative group delays as durations for wave packets shaped by purely quantum processes such as destructive interferences, which eventually comes down to questioning the principle of causality itself (*41*, *46*), see also, e.g., (*47*).

Our study thus establishes a new step toward the complete characterization of the photoemission process by providing the 3D movie of the angularly resolved dynamics of the two-photon transition leading to photoionization.

By accessing simultaneously the spectral and angular phase variations, the method allows detailed studies in both dimensions and, in particular, gives direct access to the exact photoemission delays as the local derivative of the spectral phase. Our straightforward analysis of the measured data to retrieve the complex transition amplitude is fully experimental (including the calibration procedure) and thus requires no theoretical input. This makes it applicable to a broad variety of atoms, (possibly laser-aligned) molecules, and even solid-state systems using hemispherical analyzers. The detailed information about the quantum photoemission processes will provide a stringent test for theories, in particular, aiming at describing correlated ultrafast multielectronic and vibronic dynamics, a general endeavor common to many fields. Last, the advanced characterization of resonance-enhanced multiphoton ionization provided here opens new prospects for the widely used REMPI technique (*48*) in a broad range of applications and chemical systems.

## MATERIALS AND METHODS

### Experimental setup

The experimental campaign took place on the 1-kHz attosecond beamline of the ATTOLab facility, pumped by a titanium:sapphire laser providing up to 15-mJ, 25-fs, 804-nm pulses.

The setup is shown on fig. S1. Briefly, it consists of a Mach-Zehnder interferometer in which frequency conversion is performed and two photoelectron spectrometers [a magnetic bottle electron spectrometer (MBES) and a VMIS] in a two-focus geometry (*49*, *50*). For these experiments, 3- to 5-mJ IR pulses are sent on a 90:10 beam splitter. The most intense IR beam is focused with a 2-m focal length lens in a 2-cm-long gas cell to induce HHG. The HHG process yields a comb of mutually coherent odd high harmonics in the XUV range that are first reflected on an IR-antireflective–coated silica plate that transmits most of the intense fundamental radiation. After a 200-nm Al foil that filters out the remaining IR radiation, the XUV beam is refocused into the MBES by a 500-mm focal length gold toroidal mirror at grazing incidence angle (11.5°) in a 2f-2f geometry.

The less intense IR beam, also called dressing beam, can be controlled in energy independently from the generating IR beam using an attenuator composed of a half-wave plate (λ/2) and a polarizer. The energy is set so that an intensity of ≃10^11^ W/cm^2^ is reached in the MBES and VMIS foci. The dressing delay is controlled by a piezoelectric translation stage (Piezo Jena; 5-nm resolution) (*50*). The dressing IR is then focused into the MBES using a combination of a 1140-mm lens and a 400-mm lens. This double focus enables to keep the same oddity of foci number in both arms of the interferometer, ensuring thermal drifts in same direction.

The XUV- and IR-dressing beams are recombined thanks to a drilled mirror with a 3-mm-diameter hole. Their foci are positioned in an argon effusive jet placed in the source volume of the MBES where they produce two-photon XUV-IR ionization. The MBES detection in argon [ionization potential *I*_p_(Ar) = 15.76 eV] allows for a detailed characterization of the XUV radiation, in particular, of harmonic orders 15 and 17 and of their relative phase provided by a RABBIT trace. The detection conditions in the MBES were identical in all the scans performed, allowing direct comparison of the results and spectral calibration of the VMIS data from the XUV ionizing radiation (see the Supplementary Materials). This was verified by comparing the spectral phases of the nonresonant sidebands (SB18 and SB20) measured in the MBES and in the VMIS that showed very similar linear behavior for the three scans.

Both XUV- and IR-dressing beams are then refocused by a second toroidal mirror identical to the first one in a helium gas jet in the source volume of the VMIS (*51*). This VMIS was specifically designed for attosecond spectroscopy with an original electrostatic lens and a single microchannel plate detector to maximize the spatial and angular resolutions (*52*). The measurements were taken at low repeller electrode voltage (1 kV) to maximize the resolution at low energy. Electrons with energy up to 10 eV, corresponding to SB22, were detected. When HHG was performed in xenon, all photoelectrons were detected since harmonic 21 (H21) is already in the XUV pulse cutoff. This was not the case for HHG in argon, but we verified that high-energy electrons were not polluting the measurements by recording high repeller traces that compared well to the low repeller ones.

The RABBIT scans were registered simultaneously in the MBES and VMIS over 161 steps of 50 nm, sampling 20 periods of the 400-nm sideband oscillation. Each image was accumulated over 5000 shots. Each scan took around 20 min to be registered, making the interferometer passive stability sufficient to avoid significant temporal drift during the scan. The angular distributions were obtained from the raw images by Abel inversion performed thanks to the direct algorithm for velocity-map imaging system (*53*).

### Harmonic generation conditions and results

To cover the He response over a broad range (0.85 eV), three scans were measured under three different HHG conditions corresponding to three differently blue-shifted XUV radiations. It is well known that the propagation of the laser pulse in an ionizing medium results in a blue shift of its central frequency due to the time-varying dispersion of the free electron density. This in turn results in a blue shift (and blue broadening) of the generated harmonics (*54*). The magnitude of blue shift depends on the generating gas, laser intensity, medium length, and pressure. To vary it, we played on various parameters. First, we used either argon, generating less blue-shifted harmonic combs up to H29, or xenon, enabling stronger blue shift thanks to its low ionization potential, generating harmonics up to H21. Second, we varied the intensity and phase matching conditions by playing both on a first attenuator placed before the beam splitter and on an iris placed just in front of the generation lens to control the focal diameter and energy. Third, the pressure in the 2-cm gas cell was varied as needed.

Scan (1) was taken with HHG in Ar under standard pressure and intensity conditions (the iris diameter was 17 mm and the power at the entrance of the generation chamber was 1.7 W, hence a 1.6 × 10^14^ W/cm^2^ intensity at focus); the effective generation central photon energy *E*_eff_ was 1.550 eV, which is slightly higher than the expected photon energy at 804 nm, *E*_0_ = 1.546 eV; the length of the generation medium favored blue shift even at relatively low-generation intensity. H15 had a 580-meV full width at half maximum and a central photon energy of 25.25 eV, which virtually brings the atom 150 meV above its 1s3p state. Scan (2) was taken with slightly more blue-shifted conditions, *E*_eff_ = 1.560 eV by generating in high-pressure xenon at a relatively high intensity (22-mm iris diameter, 3-W power, 4.9 × 10^14^ W/cm^2^ intensity at focus); H15 width was 640 meV. Last, scan (3) was the more blue-shifted case, *E*_eff_ = 1.578 eV with generation in high-pressure xenon and high intensity (30-mm iris diameter, 2-W power, 6.0 × 10^14^ W/cm^2^ intensity); H15 width was 730 meV.

### Simulation toolbox

All the theoretical results were obtained using a single active electron model of He. The effective electron-core potential reads, in atomic units (*55*)

$$V(r)=-\frac{1}{r}(1.23+2r)e^{-4r}$$ (5)

where *r* is the electron-nucleus distance. The first few eigen-energies of interest of this model atom, computed numerically, are reported in Table 1 and compared to the experimental ones. We see that the agreement between the two sets is very good (<35 meV).

**Table 1.** Spectroscopic data of actual and model helium atom. Relevant eigen-energies (in electron volts) of our model atom and reference values provided by the National Institute of Standards and Technology (NIST) database. The states are labeled with the corresponding configuration of the actual He. The first ionization threshold is taken as the origin of the energy scale, *E* = 0.

	**Energy (eV)**	
**State**	**Model**	**NIST (*57*)**
1s^2^	−24.588	−24.587
1s3p	−1.523	−1.491
1s4p	−0.856	−0.836
1s5p	−0.547	−0.542

The simulated photoemission data were obtained by solving the TDSE numerically with the Peaceman-Rachford algorithm (*56*) using an integration time step Δ*t* = 27 × 10^−3^ atomic units (a.u.). We chose the velocity gauge to represent the interaction between the electron and the electric fields. The temporal envelope for the XUV field is a Gaussian, while a sin^2^ shape is used for the IR field. The field characteristics (intensity and duration) were adjusted to mimic the experimental conditions, notably their intensities lying safely within the two-photon perturbative regime. A partial-wave expansion of the wave function over few (eight) angular momenta was sufficient to achieve convergence, with a radial grid extending up to *r*_max_ = 4000 a.u. by Δ*r* = 0.125 a.u. steps. We used a cos^1/8^ absorber at the edge of the grid (*56*) to prevent spurious boundary reflections of faster photoelectrons possibly released by any higher-order process. Electron spectra were computed out of the wave function upon propagation using a sliding spectral filter (window operator) with a δ*E* = 12.5 meV bandwidth (*56*).

To mimic the experimental results, three sets of TDSE simulations were run under conditions as close as possible to the experimental ones, in particular, using the same effective IR central energies (1.553, 1.560, and 1.578 eV) and XUV bandwidths. The simulated complex amplitudes were then calibrated spectrally in the same way as the experimental ones, as described in the Supplementary Materials.
