# Supplementary material for: Anisotropic dynamics of two-photon ionization: An attosecond movie of photoemission
Source: Sci Adv. 2022 Mar 23;8(12):eabl7594. doi: 10.1126/sciadv.abl7594 (PMC8942362; doi:10.1126/sciadv.abl7594)
Supplement: Supplementary file 1 — Supplementary text Figs. S1 to S8 [file sciadv.abl7594_sm.pdf]

Science Advances
AAAS

Supplementary Materials for

Anisotropic dynamics of two-photon ionization: An attosecond movie of photoemission

Alice Autuori, Dominique Platzer, Mariusz Lejman, Guillaume Gallician, Lucie Maëder, Antoine Covolo, Lea Bosse, Malay Dalui, David Bresteau, Jean-François Hergott, Olivier Tcherbakoff, Hugo J. B. Marroux, Vincent Loriot, Franck Lépine, Lionel Poisson, Richard Taïeb, Jérémie Caillat*, Pascal Salières*

*Corresponding author. Email: pascal.salieres@cea.fr (P.S.); jeremie.caillat@sorbonne-universite.fr (J.C.)

Published 23 March 2022, *Sci. Adv.* **8**, eabl7594 (2022)
DOI: 10.1126/sciadv.abl7594

The PDF file includes:

Supplementary text
Figs. S1 to S8
Legends for movies S1 and S2

Other Supplementary Material for this manuscript includes the following:

Movies S1 and S2

1. EXPERIMENTAL SETUP SCHEME

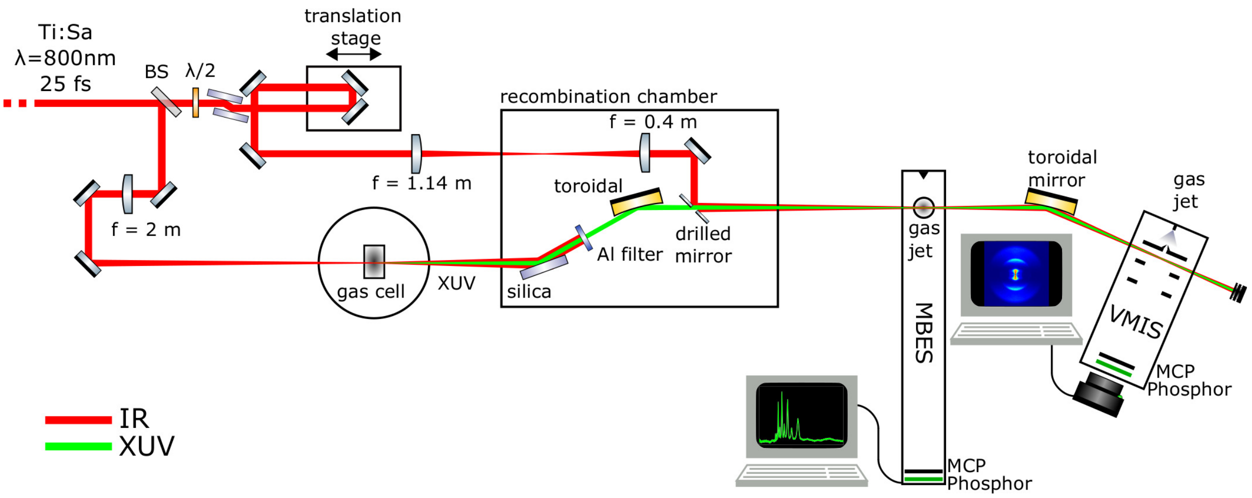

FIG. S1: **Experimental setup for spectrally- and angularly-resolved attosecond electron interferometry.**

The experimental scheme described in the ”Materials and methods” section is shown on Fig. S1.

2. RAW DATA OBTAINED AFTER RABBIT ANALYSIS

The ’raw’ data corresponding to the amplitude and phase of the Rainbow RABBIT oscillations extracted from the three scans were concatenated in Fig. S2A-B. The dotted lines define the three intervals in which each scan is shown.

Here, the spectral and angular calibration procedure detailed in Section 3 has not yet been applied to the data, including that from the spectral dependence of the ionizing radiation. The amplitudes $B_{16}(E,\theta)$ show some discontinuity between two consecutive scans, in particular between scans (1) and (2). The phases $C_{16}(E,\theta)$, being defined up to a constant independent of θ, were shifted vertically by a given amount for each scan to get the best continuity in the transition regions. Once this was done, the phase curves for the different angles showed remarkable continuity between consecutive scans. No smoothing was applied to the data, except for the phase data at angle 80 and 85° between 0.23 and 0.37 eV that are smoothed by a sliding average over 5 points for clarity around the phase jump.

The fast variations of the modulus and phase in the transition regions between resonances

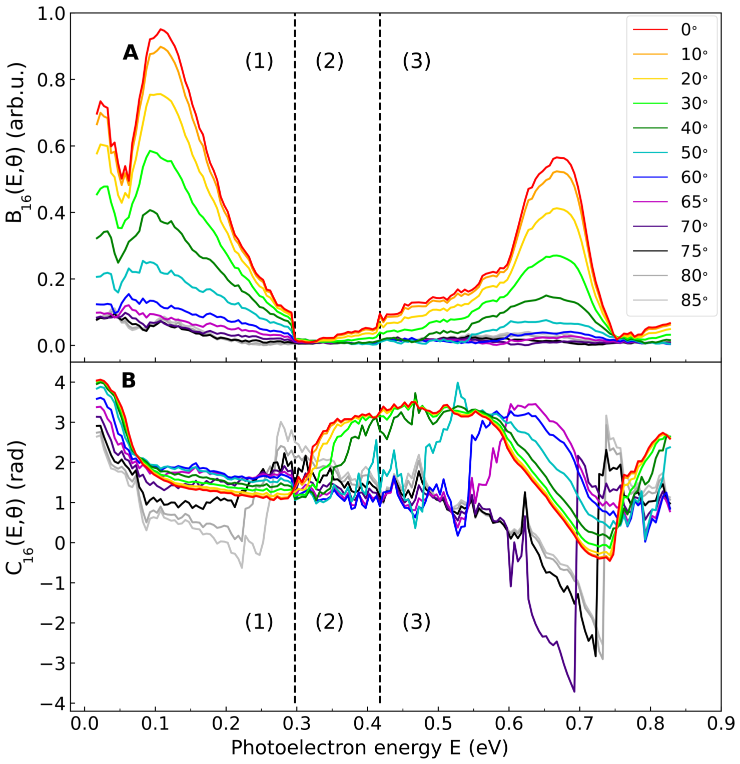

FIG. S2: **'Raw' experimental data obtained from attosecond electron interferometry.** Amplitude (**A**) and phase (**B**) of the Rainbow RABBIT oscillations extracted for the three experimental scans labeled (1), (2), (3), before the spectral and angular calibrations detailed in Section 3. The dotted lines indicate the junctions between the different scans.

allow us to estimate the spectral resolution of the VMIS. Indeed, these variations are directly due to the cancellation and change of sign of the two-photon transition amplitude. They are not smoothed by the finite (70 meV) IR spectral width which is the resolution-limiting factor in the rest of the recorded spectrum. This is clearly demonstrated by the simulations in the experimental conditions shown in Fig. S5A-B. The phase variations around $E = 0.4$ eV ($1s3p - 1s4p$ transition) and $E = 0.85$ eV ($1s4p - 1s5p$ transition) are sudden π-jumps. In the experimental data of Fig. S2B, the slope of the phase jump at $E = 0.760$ eV in scan (3) reaches +100 rad/eV for the 0° phase. This slope covers 5 points separated by 5 meV from each other, that are thus clearly distinguishable. This gives a resolution $\frac{\Delta E}{E} < 1\%$ for the VMIS at this energy, which is compatible with the expected performance of the VMIS based on simulations [52]. For the modulus in the same spectral region, the slope between 0.722 and 0.752 eV (occurring over 6 points separated by 5 meV) is particularly sharp, -6 arb.u/ eV, confirming the above estimate for the resolution.

3. FROM THE MEASUREMENTS TO THE TWO-PHOTON TRANSITION AMPLITUDE

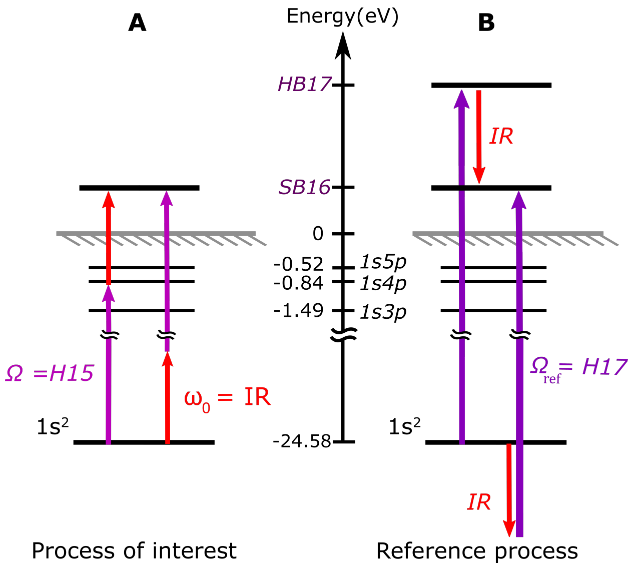

FIG. S3: **Diagrammatic representation of the photoelectron interferometric scheme.** (**A**) Process of interest. (**B**) Reference process.

We show in this section how the intrinsic transition amplitude associated with the investigated process, given by the matrix element

$$M(E,\theta) = \langle 1sE\theta|zG(\Omega)z + zG(\omega_0)z|1s^2\rangle, \tag{S1}$$

can be extracted from the angular Rainbow-RABBIT measurements. In this expression, z is the XUV and IR polarization axis, $G(\Omega)$ and $G(\omega_0)$ denote the Green operators associated with each of the sequences involved in the studied $\Omega+\omega_0$ two-photon process (see left panel of Fig. S3) [39], $|1sE\theta\rangle$ represents the $He^+(1s)+e^-(E,\theta)$ state reached by the spectrally and orientation resolved transition, and energy conservation implies:

$$E = \hbar\Omega + \hbar\omega_0 - I_p(\mathrm{He}). \tag{S2}$$

Our procedure consists in first accessing the *effective* transition amplitude, which is shaped by the XUV and IR pulse profiles, and then retrieving the intrinsic matrix element in Eq. S1 upon proper calibrations.

In terms of observables, the intensity of the detected photoelectron peak in the energy- and orientation-resolved interferometric scheme (SB16, see Fig. 1 of the main article and

Eq. S3) can be written as a function of the XUV-IR delay τ as [6, 8]

$$I_{16}(E,\theta;\tau) = A_{16}(E,\theta) + B_{16}(E,\theta)\cos[2\omega_0\tau - C_{16}(E,\theta)] \tag{S3}$$

where $A_{16}(E,\theta)$, $B_{16}(E,\theta)$ and $C_{16}(E,\theta)$ represent the average value, the $2\omega_0$ amplitude and the $2\omega_0$ phase of the SB16 oscillations when τ is varied.

The results presented in the main text are derived from the measured $B_{16}(E,\theta)$ and $C_{16}(E,\theta)$. Retrieving the fully resolved complex amplitude $M(E,\theta)$ out of these observables is equivalent to performing spectral and angular calibrations of the experiment.

A. RABBIT amplitudes and phases

The images measured in the VMIS as a function of the XUV-IR delay τ give access to the Photoelectron Angular Distribution (PAD) $I_{16}(E,\theta;\tau)$ at each sampled photoelectron energy E within SB16. This is obtained through an inverse Abel transform of the raw images calculated using Direct Algorithm for Velocity-map Imaging System [53] which projects them onto the family of Legendre polynomials $P_L(\cos\theta)$. As the interferometric scheme involves two-photon transitions, the maximal polynomial order is 4 and, as the interaction between linearly polarized pulses and atoms is independent from the azimuthal angle, the PAD for SB16 can be written as

$$I_{16}(E,\theta;\tau) = \sum_{L=0,2,4} h_L(E;\tau)P_L(\cos\theta). \tag{S4}$$

When τ is varied, each projection of the PAD onto the P_L polynomials oscillates at $2\omega_0$ frequency and can in turn be expanded as

$$h_L(E;\tau) = a_L(E) + b_L(E)\cos[2\omega_0\tau + c_L(E)] \tag{S5}$$

where the $a_L(E)$, $b_L(E)$, $c_L(E)$ parameters are determined by a Fourier analysis of $h_L(E;\tau)$ ($L = 0,2,4$). By identification, the RABBIT amplitudes $B_{16}(E,\theta)$ and phases $C_{16}(E,\theta)$ (Eq. S3) can be expressed as functions of $b_L(E)$ and $c_L(E)$:

$$B_{16}(E,\theta) = \sqrt{x(E,\theta)^2 + y(E,\theta)^2} \tag{S6}$$

$$-C_{16}(E,\theta) = \arctan\frac{y(E,\theta)}{x(E,\theta)} \tag{S7}$$

where

$$x(E,\theta) = \sum_{L=0,2,4} b_L(E)\cos c_L(E) \times P_L(\cos\theta) \tag{S8}$$

$$y(E,\theta) = \sum_{L=0,2,4} b_L(E)\sin c_L(E) \times P_L(\cos\theta). \tag{S9}$$

The amplitudes $B_{16}(E,\theta)$ and phases $C_{16}(E,\theta)$ shown in Fig. S2A-B for the three experimental scans were extracted using the above approach.

B. Link with the transition probability amplitudes

The interferometric observables (Eq. S3) can be expressed as

$$A_{16}(E,\theta) = |\mathcal{A}(E,\theta)|^2 + |\mathcal{A}_{\text{ref}}(E,\theta)|^2 \tag{S10}$$

$$B_{16}(E,\theta) = 2|\mathcal{A}(E,\theta)||\mathcal{A}_{\text{ref}}(E,\theta)| \tag{S11}$$

$$-C_{16}(E,\theta) = \arg\mathcal{A}(E,\theta) - \arg\mathcal{A}_{\text{ref}}(E,\theta) \tag{S12}$$

where $\mathcal{A}(E,\theta)$ and $\mathcal{A}_{\text{ref}}(E,\theta)$ are the *effective* amplitudes associated with the investigated process and the reference one respectively. The latter are closely related to the transition amplitudes $M(E,\theta)$ (Eq. S1) and its equivalent $M_{\text{ref}}(E,\theta)$ for the reference, but they also depend on the XUV and IR pulse characteristics [40, 41]. The extraction of $M(E,\theta)$ is made possible by considering the IR pulse ($\sim$ 70 meV width) to be monochromatic as compared to the XUV ($\gtrsim$ 500 meV width) leading to the following factorizations:

$$\mathcal{A}(E,\theta) \propto F(\Omega)F(\omega_0)M(E,\theta) \tag{S13}$$

$$\mathcal{A}_{\text{ref}}(E,\theta) \propto F(\Omega_{\text{ref}})F(\omega_0)M_{\text{ref}}(E,\theta), \tag{S14}$$

where $F(\Omega)$, $F(\Omega_{\text{ref}})$ and $F(\omega_0)$ are the field amplitudes at the XUV and IR frequencies of interest [6, 7]. Such factorization obviously does not apply when broadband pulses are used for both frequencies, as is the case for 2-photon XUV+XUV ionization where the competition between resonant and non-resonant contributions results in important distortions of the photoelectron angular distribution [43, 44]. [1]

[1] Note that the IR monochromaticity is already a prerequisite to express the RABBIT oscillations as in Eq. S3. The fact that the experimental IR has a small but non negligible width mostly results in a smoothing of the spectral features in the measurements. This has no significant consequence on their analysis and interpretation since the IR remains much narrower than the XUV, as confirmed with the simulations.

Using Eqs. S11–S14, one can express $M(E,\theta)$ as:

$$M(E,\theta) \propto \frac{1}{[M_{\text{ref}}(E,\theta)]^{\star}} \times \frac{e^{-i[\phi(\Omega)-\phi(\Omega_{\text{ref}})]}}{|F(\Omega)||F(\Omega_{\text{ref}})||F(\omega_0)|^2} \times B_{16}(E,\theta)\, e^{-iC_{16}(E,\theta)} \tag{S15}$$

where * denotes the complex conjugate, and $\phi(\Omega) = \arg F(\Omega)$ and $\phi(\Omega_{\text{ref}}) = \arg F(\Omega_{\text{ref}})$ are the phases of the XUV pulse involved in the probed and reference paths, respectively. The IR phase shift with respect to the XUV is already accounted for by the $2\omega_0\tau$ phase in Eq. S3. In Eqs. S13–S15 above, E varies concomitantly with Ω and Ω_{ref} within the support of the XUV pulse, ω_0 being fixed [see Eq. S2 and its equivalent for the reference process, $E = \hbar\Omega_{\text{ref}} - \hbar\omega_0 - I_p(\text{He})$].

As we will see in the following, accounting for the first term on the right-hand side of Eq. S15 corresponds to an angular calibration while correcting the second one corresponds to a spectral calibration of the measured amplitude B_{16} and phase C_{16}.

C. Spectral calibration

Calibration from the spectral dependence of the XUV fields

The H15 and H17 fields were characterized in the MBES. Their strengths were evaluated, up to a global factor, as

$$|F(\Omega)| \propto \sqrt{I_{15}(E' - \hbar\omega_0)} \tag{S16}$$

$$|F(\Omega_{\text{ref}})| \propto \sqrt{I_{17}(E' + \hbar\omega_0)} \tag{S17}$$

where $I_{15}(E' - \hbar\omega_0)$ and $I_{17}(E' + \hbar\omega_0)$ are the intensities of the photoelectron peaks associated with H15 and H17 obtained by photoionizing Ar in the MBES,

$$E' = E + I_p(\text{He}) - I_p(\text{Ar}) \tag{S18}$$

being the photoelectron energy in Ar corresponding to E in He. These modulus calibrating factors are shown in thick blue lines in Fig. S7.

The XUV fields phase difference is also provided by the MBES measurements in Ar, using the (angle integrated) Rainbow RABBIT technique. We show in Fig. S4A the RABBIT phase [7]

$$\Delta\varphi_{16}(E') = -\phi(\Omega) + \phi(\Omega_{\text{ref}}) + \Delta\theta_{\text{at}}(E') \tag{S19}$$

measured in the MBES for the 3 scans used in the concatenated measurements of Fig. S2. In Eq. S19, $\Delta\theta_{at}(E')$ is the so called atomic phase which essentially manifests here as a spectral bending of $\Delta\varphi_{16}(E')$ for scan (3) (green curve). This feature is a signature of the $3s^{-1}4p$ resonance in the Ar detection gas [19, 25], hit by H17 and therefore affecting SB16 around E' = 9.34 eV in Fig. S4A. Apart from this, the measured RABBIT phases evolve practically linearly. Therefore, we excluded the spurious resonant structure from our calibration procedure, and assimilated the calibration term to a linear function of energy:

$$\Delta\varphi_{16}(E') \simeq \alpha_{16}E' + \beta_{16}. \tag{S20}$$

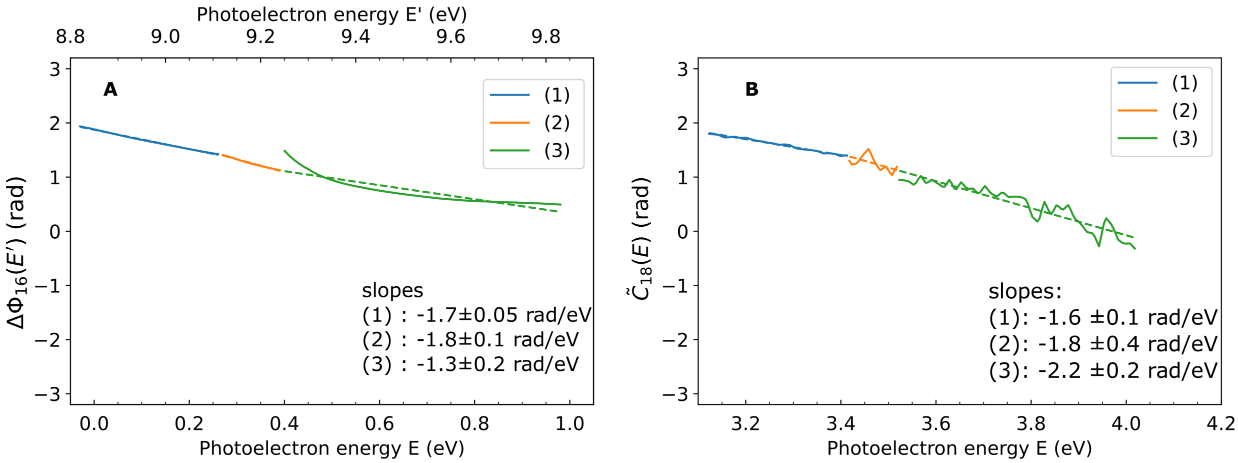

FIG. S4: **Phase measurements for the spectral calibration from the XUV fields.** Rainbow RABBIT phases measured for the three scans in Ar in the MBES at the level of SB16 (**A**) and in He in the VMIS for the angle-integrated SB18 (**B**), and corresponding slopes. The relationship between the photoelectron energies in the VMIS (E) and in the MBES (E') is given in Eq. S18.

A fit of the relevant data provides an average slope $\alpha_{16} = -1.8$ rad/eV. The probable origin of this linear phase behavior is to be found in the intrinsic harmonic chirp that induces a quadratic spectral phase in each harmonic line. In absence of blue-shift, the very similar phase evolution of consecutive harmonics results in a roughly constant sideband phase. In presence of blue-shift of the generating IR beam, but not of the dressing IR beam, the sideband phase presents a linear behavior with a slope proportional to the amount of frequency shift [24]. The increase of the measured slope with increasing blue-shift is another indication of this phenomenon.

In order to highlight the consistency of the measurements performed simultaneously in the VMIS on the one hand and in the MBES on the other hand, we also performed a Rainbow RABBIT analysis of the unstructured SB18 detected with the VMIS in He, that involves two non-resonant transitions. The resulting phase $C_{18}(E,\theta)$ – the equivalent of $C_{16}(E,\theta)$ for the SB18 peak, is angle integrated and shown in Fig. S4B for the three concatenated scans. It varies linearly, with a slope very close to α_{16} measured in the MBES. This therefore reinforces the validity of our spectral phase calibration procedure.

Calibration from the spectral dependence of the reference transition amplitude

Since the reference process is a two-photon transition through a smooth continuum, both $|M_{\mathrm{ref}}(E,\theta)|$ and $\arg M_{\mathrm{ref}}(E,\theta)$ can be considered as *spectrally* constant within the SB16 width. This assumption is validated by the results of simulations in Fig. S5C-D, showing slight spectral variations of the modulus and a constant phase. It is furthermore comforted experimentally by the negligible spectral variations (except for the linear behavior discussed above) displayed in Fig. S4B by the angle-integrated $C_{18}(E,\theta)$.

At this point, the studied transition amplitude can thus be written as

$$M(E,\theta) = \mathcal{D}(\theta) \times \frac{e^{i\alpha_{16}E'}}{\sqrt{I_{15}(E' - \hbar\omega_0) I_{17}(E' + \hbar\omega_0)}} \times B_{16}(E,\theta)\, e^{-iC_{16}(E,\theta)}. \tag{S21}$$

The modulus of the spectrally constant factor $\mathcal{D}(\theta)$ encompasses the IR field strength, the implicit proportionality factors of Eqs. S15, S16 and S17 and the modulus of the reference amplitude. Its phase encompasses the harmonic phase offset, which is here irrelevent, and the spectral phase of the reference transition amplitude. The θ dependence of $\mathcal{D}(\theta)$, addressed in the following section, is therefore inherited from the reference transition amplitude $M_{\mathrm{ref}}(E,\theta)$ solely.

D. Angular calibration

We tackle here the angular calibration of the measured data and hence complete the procedure that gives full access to $M(E,\theta)$ (up to a constant factor) out of exclusively experimental data. The simulated data shown in Fig. S5C-D evidence some significant angular variations of the (17-1) reference transition amplitude: Its phase decreases smoothly by 0.7

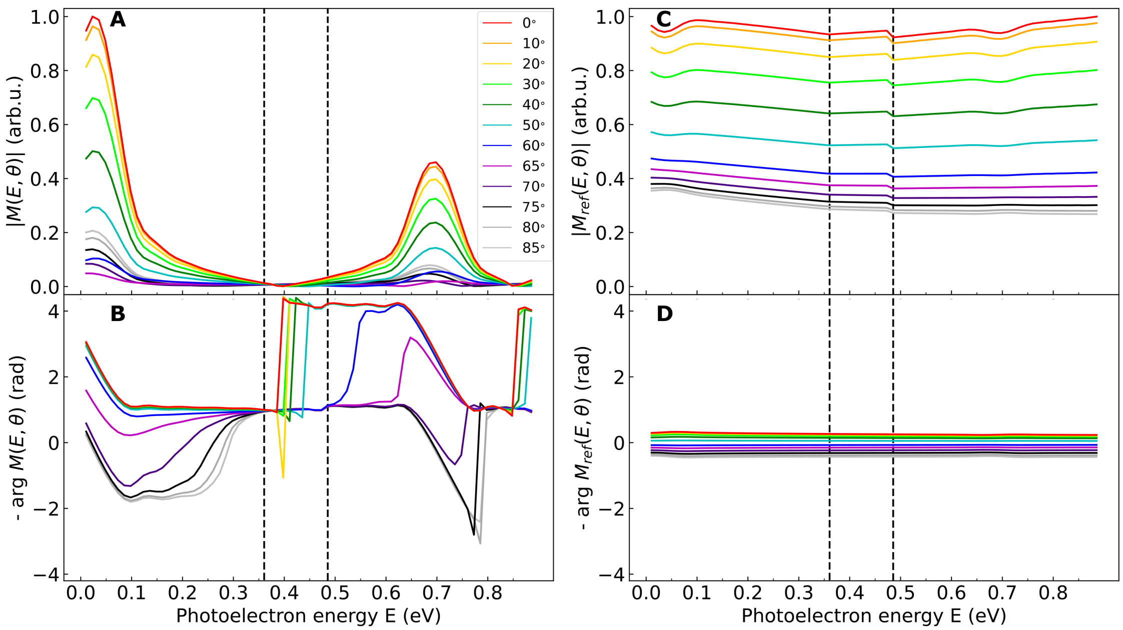

FIG. S5: **Simulation of the investigated and reference complex transition amplitudes.** Simulated modulus and phase of the investigated transition amplitude (**A** and **B**), and of the reference transition amplitude (**C** and **D**) across the SB16 spectral region for angles between 0 and 85°. Vertical dotted lines: limits of the concatenations, equivalent to the ones performed in the experiments (see Materials and methods).

rad over 90° and its amplitude is divided by 3 in the same interval. This is consistent with the Fano propensity rule extended to two-photon transitions [37] that favors the s final state over the d state in the case of stimulated emission of the IR photon, but in a proportion here that is not sufficient to eliminate completely the angular dependence of the modulus and phase of the reference transition.

Calibration from the angular dependence of the reference transition amplitude

Our angular calibration procedure relies on the partial wave decomposition of the experimental data introduced in [33]. The high structuration of SB16 imprinted by the investigated transition however prevents from efficiently applying this procedure to directly extract the relative amplitudes and phases of the s and d contributions of the (17-1) reference path. Nevertheless, a tractable bypass consists in exploiting the neighboring SB18 peak. Indeed, the amplitude of the (19-1) transition which contributes to the latter peak is likely to display angular variations very close to the ones of the reference transition amplitude.

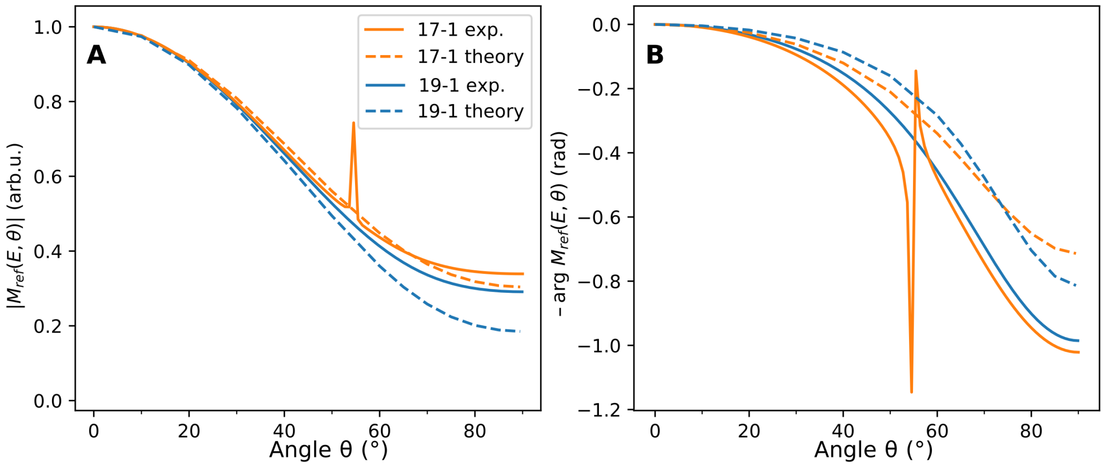

FIG. S6: **Comparison of the experimental and simulated reference transition amplitudes.** Experimental (solid) and theoretical (dashed) angular dependence of the modulus (**A**) and phase (**B**) of the (17-1) reference transition amplitude (orange) and of its (19-1) surrogate (blue).

As detailed in [33], the s and d components of each path leading to SB18 can be retrieved by fitting the oscillations of the $h_L(\tau)$ functions (introduced in Eq. S4 for SB16). SB18 was spectrally integrated over its ≈ 500-meV width before performing the multi-parameter fit in order to ensure a satisfactory signal-to-noise ratio. The fit provides the transition amplitudes $M_{17+1}(E,\theta)$ and $M_{19-1}(E,\theta)$, as well as the corresponding partial wave components $\mathcal{M}^{(s)}_{17+1}$, $\mathcal{M}^{(d)}_{17+1}$ and $\mathcal{M}^{(s)}_{19-1}$, $\mathcal{M}^{(d)}_{19-1}$, respectively. Due to the Fano propensity rules extended to two-photon transitions [37], $M_{17+1}(E,\theta)$ exhibits a node and change of sign at angle $\theta = 67.3°$, which is not the case for $M_{19-1}(E,\theta)$. Their phase difference, encoded in the RABBIT phase, is thus angularly dependent [29, 37], which should not be interpreted in terms of 'angular variation of the photoemission delay'. This highlights the irrelevance of the standard expression of the atomic delay, $\tau_A(E,\theta) = [\arg M_{19-1}(E,\theta) - \arg M_{17+1}(E,\theta)]/2\omega_0$ to represent the ionization dynamics in this context.

We thus get access to the angular dependence of $M_{19-1}(E,\theta)$, acting as a surrogate of the reference transition amplitude, leading to:

$$\mathcal{D}(\theta) \propto \frac{1}{[M_{\text{ref}}(E,\theta)]^{\star}} \propto \frac{1}{[\mathcal{M}^{(s)}_{19-1}/\mathcal{M}^{(d)}_{19-1}]^{\star} \times Y_{00}(\theta) + Y_{20}(\theta)}. \tag{S22}$$

Note that only the ratio of the partial amplitudes plays a role in Eq S22.

Eventually, both spectral and angular calibrations relate the sought-for transition amplitudes to the experimental data as:

$$M(E,\theta) = \mathcal{N} \times \frac{1}{[\mathcal{M}^{(s)}_{19-1}/\mathcal{M}^{(d)}_{19-1}]^\star + \sqrt{5/4}\,(3\cos^2\theta - 1)} \times \frac{e^{i\alpha_{16}E'}}{\sqrt{I_{15}(E'-\hbar\omega_0)I_{17}(E'+\hbar\omega_0)}}$$
$$\times B_{16}(E,\theta)\, e^{-iC_{16}(E,\theta)} \text{(S23)}$$

where $\mathcal{N}$ is a spectrally and angularly constant factor. The data displayed and discussed in the main paper (Fig. 2) were obtained with this formula.

Assessing the angular calibration

The relevance of SB18 as a surrogate peak to access the θ-dependence of the reference transition amplitude is highlighted in Fig. S6. It evidences the excellent agreement between the angular variation of the experimental (19-1) transition amplitude (blue solid curve) with both i) the theoretical equivalent (blue dotted curve) and ii) the angular variation of the simulated (17-1) reference amplitude (orange dashed curve).

To further assess the robustness of our angular calibration, which is a major novelty of our study and of paramount importance for the *complete* characterization of the investigated photoemission process, we cross checked it in an independent way. In contrast to the calibration procedure presented above, the present assessment uses some *a priori* knowledge on the investigated process to reconstruct the angular dependence of the reference amplitude.

Having in mind that the probed transition ends up in a combination of s and d partial waves, we identified in the experimental data of Fig. S2 the energy $E_0^{(s)}$ where the s component is canceled due to opposite contributions from the two neighboring $1s3p$ and $1s4p$ resonances (see Eq. 3 in main article). At this energy, we expect a $\approx \pi$ rad phase jump to occur at the nodal angle of $Y_{20}(\theta)$, $\theta_m \simeq 54.7°$. This provides the experimental value $E_0^{(s)} = 0.547$ eV, in excellent agreement with the position given by the experimental partial wave decomposition in Fig. 3A-B in manuscript. The equivalent cancellation in the simulated data shows up at 0.47 eV in Fig. 3C-D.

At this energy, $M(E_0^{(s)},\theta)$ is thus shaped by $Y_{20}(\theta)$, and the remaining angular dependence in $B_{16}(E_0^{(s)},\theta)$ and $C_{16}(E_0^{(s)},\theta)$ is ascribed to $M_{\mathrm{ref}}(E_0^{(s)},\theta)$. In order to access the latter, it is therefore sufficient (see Eq. S15) to correct the former by $Y_{20}(\theta)$. The angular factor $\mathcal{D}(\theta)$ (Eq. S21) can thus be retrieved as:

$$\mathcal{D}(\theta) \propto \frac{1}{[M_{\mathrm{ref}}(E_0^{(s)},\theta)]^\star} \propto Y_{20}(\theta) \times \frac{e^{+iC_{16}(E_0^{(s)},\theta)}}{B_{16}(E_0^{(s)},\theta)}. \quad \text{(S24)}$$

This angular dependence, evaluated here at the specific cancellation energy $E_0^{(s)}$, is furthermore expected to apply identically at all E within SB16, as already mentioned above. The right hand side of Eq. S24 is displayed in Fig. S6 (orange solid curve), together with the simulated (17-1) transition amplitude (orange dashed curve). Apart from numerical outliers around the nodal angle θ_m, we find an excellent agreement between the two curves. They are moreover very close to the experimental and simulated angular dependencies for the (19-1) reference (blue curves). This complementary analysis hence strengthens the relevance of the (19-1) transition as a surrogate reference for the angular calibration of our experiments.

The final result of the calibration, i.e., $M(E,\theta)$, is compared in Fig. S7 to the 'raw' data of the amplitude $B_{16}(E,\theta)$ and phase $C_{16}(E,\theta)$ of the Rainbow RABBIT oscillations (see Fig. S2). The original data clearly displays the essential of the spectral and angular structures of the intrinsic transition amplitude, which highlights the relevance of the (17-1) transition as an appropriate reference inducing weak distortions that can be calibrated.

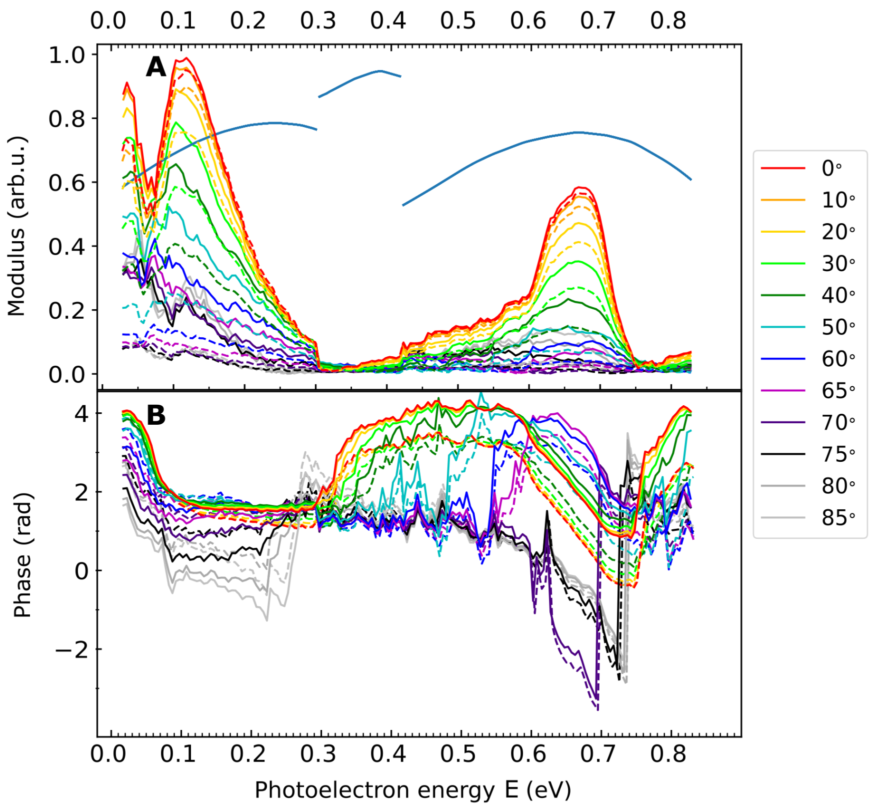

FIG. S7: **Outcome of the spectral and angular calibrations.** Comparison of the retrieved intrinsic transition amplitude $M(E,\theta)$ (solid lines) with the initial 'raw' Rainbow RABBIT data (dashed lines): (**A**) $|M(E,\theta)|$ and $B_{16}(E,\theta)$ moduli; (**B**) -arg $M(E,\theta)$ and $C_{16}(E,\theta)$ phases. The thick blue curves in (A) represent the factor $\sqrt{I_{15}I_{17}}$ as measured in the MBES for each scan (1)-(3), introduced in Eq. S21 for the spectral calibration from the XUV field amplitude.

4. PHASE MEASUREMENT UNCERTAINTY

The uncertainty on the $C_{16}(E,\theta)$ phases can be calculated from the uncertainty on the amplitudes b_L and phases c_L of the $2\omega_0$ oscillations of the $h_L(E;\tau)$ functions (see Eq. S5). From the derivation of Eq. S7, the uncertainty writes

$$\Delta C_{16}(E,\theta) = \sum_L \Bigg[|x(E,\theta)\sin c_L(E) - y(E,\theta)\cos c_L(E)| \frac{|P_L(\cos\theta)|}{x(E,\theta)^2 + y(E,\theta)^2} . \Delta b_L(E) + |x(E,\theta)\cos c_L(E) + y(E,\theta)\sin c_L(E)| \frac{|b_L(E) P_L(\cos\theta)|}{x(E,\theta)^2 + y(E,\theta)^2} . \Delta c_L(E) \Bigg] \quad \text{(S25)}$$

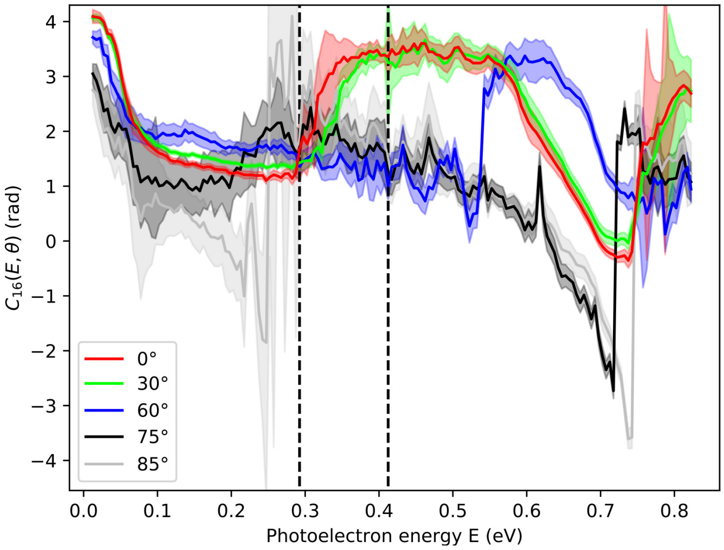

FIG. S8: **Evaluation of the uncertainty on the phase measurements.** Phase of the Rainbow RABBIT oscillations extracted for the three experimental scans (separated by dotted lines) and corresponding uncertainty for five different angles θ.

where the first line comes from the uncertainty on the amplitudes and the second line from the uncertainty on the phases. The $\Delta b_L(E)$ and $\Delta c_L(E)$ uncertainties were determined from the $2\omega_0$ peak signal to noise ratio in the Fourier transform analysis.

Figure S8 displays the phase of the Rainbow RABBIT oscillations $C_{16}(E,\theta)$ and the corresponding uncertainty for 5 angles. At small angle, the phase error remains low, except at the position of the sudden phase jumps in the transition regions where the transition amplitude cancellations lead to a low signal to noise ratio. The error slowly increases with angle but remains below 300 meV, except at very high angles, 80 and 85° in scan (1), where the error diverges at the phase jumps. This is why the corresponding phase curves shown in Fig. S2B (and resulting curves for the transition amplitude in the manuscript) are smoothed via a 5 points sliding average in the 0.227-0.367 eV spectral region. In general, as the uncertainty is calculated from the partial wave coefficients (who are determined over the whole projected Newton sphere by Abel transform, therefore carrying low error themselves), the error remains reasonably small even at high angles where the signal is low.

5. COMPLETE DYNAMICS RECONSTRUCTION

In complement to the θ-dependent transition delay τ_{tran} (Eq. 4 and Fig. 4A of the manuscript), complete insight into the photoemission dynamics was obtained with the gated inverse Fourier transform:

$$\hat{M}(t,E;\theta) = \frac{1}{\hbar}\int_{-\infty}^{+\infty} d\varepsilon\, M(\varepsilon,\theta)\; e^{-(\varepsilon-E)^2/2\Delta^2}\; e^{-i\varepsilon t/\hbar} \tag{S26}$$

of the transition amplitudes $M(E,\theta)$ shown in Fig. 2 of the manuscript. This spectro-temporal amplitude was used to reconstruct the dynamics revealed by Figs. 4B-D and Movies S1 and S2. We set the spectral width of the sliding Gaussian filter to $\Delta = 90$ meV (corresponding to 210 meV FMHM) to get a balanced trade-off between the time and spectral resolutions.

A. Caption for angular movie S1: Experimental reconstruction of the spectro-temporal dynamics of helium photoionization as a function of electron emission angle.

Movie S1 displays the Gabor representation of the ionization amplitude of helium as a function of electron emission angle. It corresponds to $|\hat{M}(t,E;\theta)|^2$ (see Eq. S26) experimentally reconstructed in the spectral region of interest, $0 < E < 0.85$ eV. The movie covers angles ranging from 0 to 89.5° with 0.5° steps. The intensity was renormalized at each angle in order to keep the same colorscale along the movie.

The spectro-temporal buildup of the ionization process is strongly shaped by the presence of the intermediate resonances $1s3p$ and $1s4p$ at 0.06 and 0.66 eV respectively. Furthermore, the buildup changes dramatically for angles above 50° and photoelectron energies above 0.3 eV due to the angularly-varying spectral phase jumps in the transition region between the resonances.

B. Caption for temporal movie S2: Experimental and theoretical reconstructions of the angularly-resolved temporal dynamics of helium photoemission in real time.

Movie S2 displays the temporal evolution of the complex ionization amplitude of helium in the transition region between the $1s3p$ and $1s4p$ intermediate resonances. It is obtained by reconstructing $\hat{M}(t,E_0;\theta)$ (Eq. S26) using a 210 meV-Gaussian filter centered at photoelectron energy $E_0 = 0.38$ eV for the experimental frame and $E_0 = 0.40$ eV for the theoretical

one. The movie displays the modulus $|\hat{M}(t, E_0; \theta)|$ in polar representation, while the phase $\arg \hat{M}(t, E_0; \theta)$ is given by the color scale. The origin of time for both the experimental and theoretical reconstructions is fixed at the center of the (isotropic) temporal profile of SB18, previously calibrated from the linear spectral phase $\alpha_{16} E'$ in the experimental case (see Fig. S4B and Eq. S20).

The ionization amplitude exhibits important temporal modulations due to the strong spectral and angular variations in the transition region between the $1s3p$ and $1s4p$ resonances. In particular, the spectral phase jumps induce a destructive temporal interference around the time origin associated with a strong change of shape of the emission dynamics. In contrast, the angular distributions around the temporal maxima before and after the destructive interference ($t \approx -5.8$ fs and $+7.9$ fs in the experiment) are very stable, with similar shapes but π-rad shifted phases.
